# Increased medial component gap at 30° flexion is associated with subjective instability and worse patient‐reported outcomes after image‐free robot‐assisted total knee arthroplasty

**DOI:** 10.1002/jeo2.70865

**Published:** 2026-07-23

**Authors:** Keisuke Maeda, Tomoharu Mochizuki, Keiichiro Someya, Yutaka Fujita, Yuya Majima, Shigeru Takagi, Go Omori, Hiroyuki Kawashima

**Affiliations:** ^1^ Division of Orthopedic Surgery Niigata University Medical and Dental Hospital Niigata Japan; ^2^ Department of Orthopedic Surgery Niigata Rehabilitation Hospital Niigata Japan; ^3^ Department of Health and Sports Niigata University of Health and Welfare Niigata Japan

**Keywords:** component gap, component positioning, mid‐flexion instability, patient‐reported outcomes, robot‐assisted total knee arthroplasty

## Abstract

**Purpose:**

Instability after total knee arthroplasty (TKA), particularly in the mid‐flexion range, is associated with reduced patient satisfaction and poorer functional outcomes. Image‐free robot‐assisted TKA (rTKA) enables quantitative intraoperative assessment and adjustment of soft‐tissue balance and component position; however, it remains unclear which intraoperative component gap (CG) at each flexion angle and in each compartment is most strongly associated with postoperative outcomes and which component‐positioning factors determine that gap.

**Methods:**

This prospective cohort study included 72 consecutive knees that underwent primary image‐free rTKA using the Journey II Bi‐Cruciate Stabilised knee system between 2021 and 2024. Intraoperative CG was measured at 0°, 30°, 60° and 105° of flexion using an independently tensioned medial‐lateral ligament tensioner and was defined as the measured gap minus the thickness of the final insert used. Clinical assessment was performed preoperatively and at 1 year postoperatively using self‐reported knee instability (SRKI) and the knee injury and osteoarthritis outcome score (KOOS). Associations of each CG with SRKI and KOOS total at 1 year were examined using regression analyses adjusted for age and body mass index (BMI). In addition, the CG associated with worse postoperative clinical outcomes was used as the dependent variable to analyse its associations with component‐positioning factors.

**Results:**

Both medial and lateral CGs were significantly smaller at 30° and 60° of flexion than at 0° and 105°. Among all measured gaps, the medial CG at 30° of flexion (Med CG30°) showed the most consistent association with postoperative outcomes. An increase in Med CG30° was significantly associated with SRKI positivity at 1 year and with a lower KOOS total score. In multivariable analysis, Med CG30° was independently associated with femoral coronal, sagittal and rotational alignment, whereas no significant associations were found with tibial component‐positioning factors.

**Conclusion:**

In image‐free rTKA using the Journey II system, an increased medial CG at 30° of flexion was associated with postoperative subjective instability and worse patient‐reported clinical outcomes. In soft‐tissue balance assessment during TKA, attention should be paid not only to the extension and flexion gaps but also to the Med CG30°.

**Level of Evidence:**

Level II.

## Graphical abstract

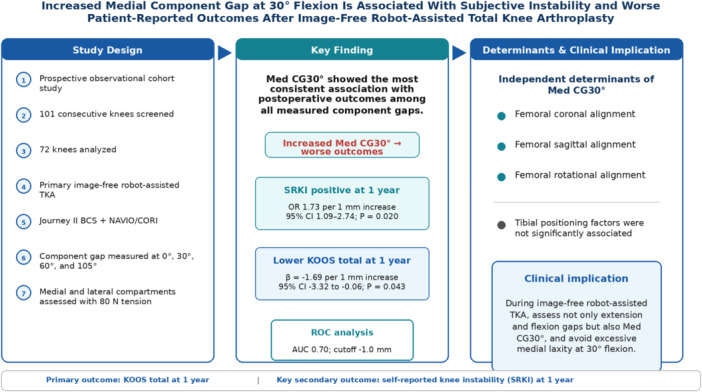

**Abbreviations**
ADL: activities of daily living
AUC: area under the curve
BMI: body mass index
CG: component gap
CI: confidence interval
HKA: hip‐knee‐ankle angle
KOOS: Knee injury and Osteoarthritis Outcome Score
MFI: mid‐flexion instability
OR: odds ratio
QOL: quality of life
ROC: receiver operating characteristic
rTKA: robot‐assisted total knee arthroplasty
SEA: surgical epicondylar axis
SRKI: self‐reported knee instability
TKA: total knee arthroplasty

## INTRODUCTION

Although the long‐term results of total knee arthroplasty (TKA) have improved in recent years [3, 16, 24], instability remains one of the leading causes of revision in the short to mid‐term and is also associated with lower patient satisfaction and poorer functional outcomes after surgery [9, 17, 18, 29]. Instability has been reported to account for approximately 11.6% of all revision TKAs and 18.4% of early revision procedures [17, 18]. Therefore, identifying intraoperative factors associated with postoperative subjective instability may improve patient outcomes and reduce the risk of revision surgery. In particular, instability from extension to the mid‐flexion range, so‐called mid‐flexion instability (MFI), has attracted increasing attention and multiple factors have been considered to contribute to its development, including changes in the joint line, implant design, amount of bone resection, soft‐tissue balance and component position [5, 8, 11, 13, 19, 21, 22, 25].

In recent years, robot‐assisted TKA (rTKA) has become increasingly used because, compared with conventional techniques and standard navigation, it enables more accurate bone resection and implant positioning and allows optimisation of soft‐tissue balance by adjusting component placement intraoperatively [4, 6, 10, 23]. In particular, image‐free rTKA is characterised by reconstruction of joint morphology through intraoperative mapping without reliance on preoperative imaging, allowing component placement to be adjusted in six degrees of freedom. This makes it possible to proceed with surgery while quantitatively evaluating alignment and soft‐tissue balance and to plan reconstruction while considering restoration of the joint line, including the articular cartilage [20]. Because of these characteristics, image‐free rTKA is expected to help improve MFI.

Previous research has shown that, in image‐free rTKA using the Journey II implant, the component gap (CG) in the mid‐flexion range was smaller than that in extension and deep flexion and might contribute to stabilisation in the mid‐flexion range [20]. However, that study focused primarily on the distribution of intraoperative CGs and changes in the joint line, and it did not clarify how differences in intraoperative CGs were related to actual postoperative patient‐reported clinical outcomes.

To better understand instability after TKA, it is important to determine which gap laxity among the CGs at each flexion angle and in each compartment is most strongly associated with postoperative outcomes. Furthermore, identifying the component‐positioning factors that determine the CG associated with postoperative outcomes is clinically meaningful for optimising intraoperative planning and improving postoperative results. Therefore, the purpose of this study was to investigate the associations between intraoperative CG at each flexion angle and in each compartment and postoperative clinical outcomes after image‐free rTKA using the Journey II system, and to analyse the component‐positioning factors that determine the CG associated with worse postoperative clinical outcomes.

The study hypothesis was that increased medial CG at 30° flexion would be associated with subjective knee instability and worse patient‐reported outcomes.

## MATERIALS AND METHODS

### Study population

This prospective observational cohort study of consecutive patients was approved by the institutional ethics committee, and written informed consent was obtained from all participants. Between April 2022 and March 2024, consecutive patients with varus knee osteoarthritis who underwent primary image‐free rTKA using the Journey II Bi‐Cruciate Stabilised knee system (Smith & Nephew) with either the NAVIO® or CORI® robotic system were screened.

The inclusion criteria were patients with varus knee osteoarthritis who underwent primary image‐free rTKA using the Journey II Bi‐Cruciate Stabilised knee system and had available intraoperative CG data, postoperative CT‐based component‐position assessment and 1‐year clinical outcomes. The exclusion criteria were valgus knee deformity, flexion contracture greater than 20°, previous high tibial osteotomy, medial collateral ligament repair, intraoperative robotic system malfunction requiring conversion to manual instrumentation, missing postoperative CT data, loss to follow‐up or follow‐up at another institution and early postoperative fracture.

### Surgical technique

All procedures were performed by three experienced surgeons using a standardised surgical technique through a medial parapatellar approach. An image‐free semi‐active robotic system, either NAVIO® or CORI®, was used in all cases. These systems do not require preoperative CT or MRI and can construct a three‐dimensional model of the knee joint through intraoperative mapping, allowing real‐time implant planning, alignment assessment and gap assessment. For preoperative planning, three‐dimensional software (JIGEN®; LEXI, Inc.) was used to determine femoral and tibial component size and the initial component position. The initial femoral component position was set at 0° relative to the mechanical axis in the coronal plane, 3° of flexion in the sagittal plane and 0° relative to the surgical epicondylar axis (SEA) in rotation. The initial tibial component position was set at 0° relative to the mechanical axis in the coronal plane, 3° of posterior slope in the sagittal plane and rotation referenced to the Akagi line or ROM technique [4].

Intraoperatively, the femoral component position was fine‐tuned while considering overall limb alignment and soft‐tissue balance. The allowable adjustment range for the femoral component was 0 ± 3° in the coronal plane, 0 ± 6° in the sagittal plane and ±3° relative to the SEA in rotation. In contrast, the tibial component was basically fixed at 0° in the coronal plane and 3° of posterior slope in the sagittal plane, with rotation determined according to the aforementioned references. Before gap assessment, osteophytes were removed and the posterior cruciate ligament was resected in all cases because a bi‐cruciate stabilised insert was used. Minimal medial soft‐tissue release was performed as needed, whereas lateral release was not performed. Additional soft‐tissue releases were not routinely performed. The target soft‐tissue balance was −2 to 0 mm medially and 0 to 5 mm laterally in extension and −1 to 3 mm medially and 0 to 5 mm laterally in flexion. The tibial component position was maintained according to the preoperative plan, and the femoral component position was adjusted within the predefined femoral adjustment ranges described above to achieve the target gap balance as closely as possible.

### Intraoperative measurement of CG

After femoral and tibial bone resections, a femoral trial component was placed and CG was measured using an independently tensioned medial‐lateral ligament tensioner [12, 20]. Measurements were obtained at 0°, 30°, 60° and 105° of knee flexion, with 80 N of distraction force applied to both the medial and lateral compartments. During measurement, the patellofemoral joint was reduced. Because the Journey II system recommends flexion‐gap evaluation at 105° to correspond to the five‐in‐one femoral resection, flexion CG was assessed at 105° in this study as well. CG was defined as the measured medial or lateral gap at each flexion angle minus the thickness of the final insert used. Negative values indicated a tighter gap, whereas positive values indicated a looser gap.

### Evaluation of component positioning

**Figure 1:**
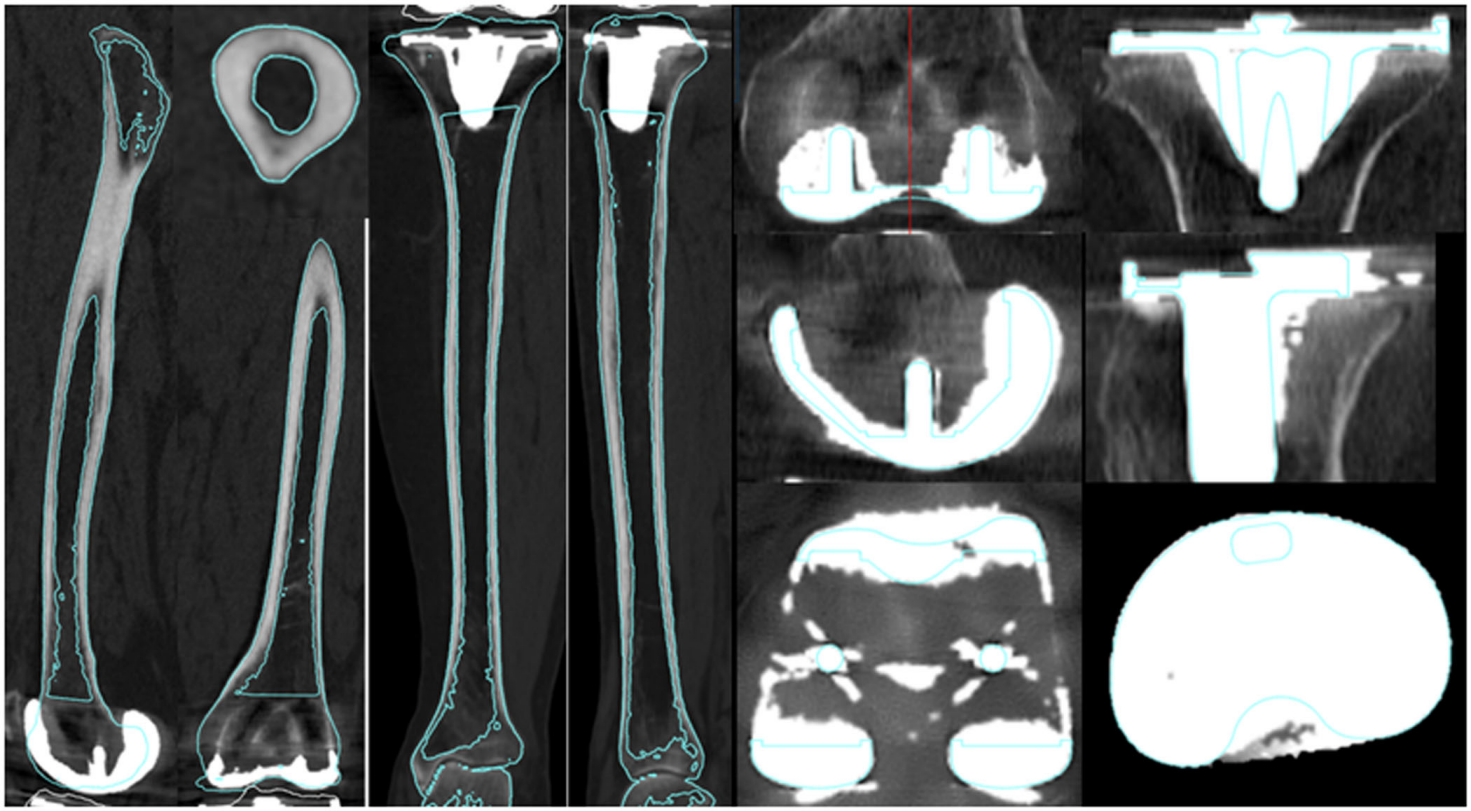
Evaluation of component position using three‐dimensional matching.

Component position was evaluated using preoperative and postoperative full‐length lower‐extremity CT data. Preoperative CT scans were used for preoperative implant positioning planning, whereas postoperative CT scans obtained 2 weeks after surgery were used for assessment of postoperative implant positioning. Preoperative three‐dimensional bone models and postoperative three‐dimensional models (bone plus components) were created and embedded with the same anatomical coordinate system [28], after which pre and postoperative three‐dimensional matching was performed (Figure 1). Component alignment was assessed using JIGEN® software (LEXI Co., Ltd.). JIGEN® utilises preoperative lower‐extremity CT data for three‐dimensional surgical planning and postoperative CT‐based three‐dimensional assessment of component position through three‐dimensional image matching. The evaluated parameters were femoral coronal alignment, femoral sagittal alignment, femoral rotational alignment, tibial coronal alignment, tibial sagittal alignment and tibial rotational alignment. For the femur, valgus was defined as positive and varus as negative in the coronal plane, extension as positive and flexion as negative in the sagittal plane and external rotation as positive and internal rotation as negative in the axial plane. The same sign conventions were used for the tibia.

### Clinical evaluation

Clinical assessment was performed preoperatively and at 1 year postoperatively. The primary outcome was the KOOS total score at 1 year postoperatively. KOOS is a validated self‐administered patient‐reported outcome measure that assesses pain, symptoms, activities of daily living, sport and recreation function and knee‐related quality of life [26]. In this study, all KOOS scores were converted to a 0–100 scale.

SRKI at 1 year was evaluated as a key secondary outcome because the purpose of this study was to investigate the relationship between intraoperative CG measurements and subjective instability after rTKA. Subjective knee instability was assessed according to the method described by van der Esch et al. [7] using a patient‐reported instability question. Patients were asked whether they experienced subjective knee instability, including buckling, shifting or giving way during daily activities. Knees were classified as SRKI‐positive when subjective instability was reported and SRKI‐negative when instability was not reported.

In addition to changes in clinical outcomes from before to after surgery, the associations of KOOS and SRKI at 1 year with intraoperative CG and component position were analysed.

### Statistical analysis

Continuous variables are presented as mean ± standard deviation, and categorical variables as number and percentage. Preoperative and 1‐year postoperative continuous variables were compared using paired *t*‐tests, and SRKI was analysed as a dichotomous variable (positive or negative). The exact McNemar test was used to compare paired proportions between preoperative and postoperative assessments because of the relatively small number of SRKI‐positive cases. To examine associations between CG at each flexion angle and in each compartment and postoperative clinical outcomes, logistic regression analysis was used when SRKI was the dependent variable, and linear regression analysis was used when KOOS total was the dependent variable; both models were adjusted for age and body mass index (BMI). Effect sizes are reported per 1‐mm increase in CG. Receiver operating characteristic (ROC) analysis was then performed for the CG associated with postoperative clinical outcomes to evaluate discrimination for SRKI, and the area under the curve (AUC) and optimal cutoff value were calculated. In addition, associations with each component‐positioning factor were first examined using univariable linear regression analysis, followed by multivariable linear regression analysis including femoral and tibial coronal, sagittal and rotational alignment simultaneously. Regression coefficients (*β*) are reported per 1° increase in each positioning angle. Statistical analyses were performed using SPSS, with statistical significance set at *p* < 0.05.

A power analysis was performed based on the association between CG and KOOS total. Assuming a moderate correlation (*r* = 0.35), *α* = 0.05, and power = 0.80, a minimum sample size of 62 knees was required. Therefore, the final sample size of 72 knees was considered adequate.

## RESULTS

A total of 101 consecutive knees were enrolled during the study period. No eligible patients declined participation. Twenty‐nine knees were excluded from the final analysis for the following reasons: loss to follow‐up or follow‐up at another institution (*n* = 9), flexion contracture greater than 20° (*n* = 6), intraoperative robotic system malfunction requiring conversion to manual instrumentation (*n* = 5), medial collateral ligament repair (*n* = 2), missing postoperative CT data (*n* = 2), valgus knee deformity (*n* = 3), previous high tibial osteotomy (*n* = 1) and early postoperative fracture (*n* = 1). Consequently, 72 knees were included in the final analysis. Baseline demographic and clinical characteristics are summarised in Table 1.

**Table 1:** Demographic data.

Variable	Overall (*n* = 72)
Number of knees	72
Sex (female/male)	63/9
Age, years	73.4 ± 6.7
BMI, kg/m^2^	25.9 ± 5.4
Preoperative HKA, °	190.6 ± 5.5
Preoperative extension, °	−5.9 ± 5.4
Preoperative flexion, °	123.8 ± 15.9

*Note*: Data are expressed as mean ± standard deviation.

Abbreviations: BMI, body mass index; HKA, hip‐knee‐ankle angle.

### Preoperative and 1‐year postoperative clinical outcomes

Clinical outcomes improved significantly at 1 year postoperatively compared with preoperative values (Table 2).

**Table 2:** Comparison of preoperative and postoperative SRKI and KOOS scores.

Variable	Preoperative	Postoperative at 1 year	*p‐*Value
SRKI positive, *n* (%)	32 (44.4%)	12 (16.7%)	<0.001
KOOS total	52.8 ± 17.3	75.1 ± 12.4	<0.001
KOOS symptoms	63.9 ± 20.2	81.7 ± 15.4	<0.001
KOOS stiffness	56.1 ± 27.0	78.8 ± 18.6	<0.001
KOOS pain	53.0 ± 19.4	83.4 ± 13.8	<0.001
KOOS ADL	62.1 ± 18.5	80.7 ± 12.0	<0.001
KOOS sports/recreation	23.8 ± 22.7	42.7 ± 25.5	<0.001
KOOS QOL	33.9 ± 20.9	62.9 ± 20.9	<0.001

*Note*: Data are expressed as mean ± standard deviation.

Abbreviations: ADL, activities of daily living; KOOS, knee injury and oisteoarthritis outcome score; QOL, quality of life; SRKI, self‐reported knee instability.

### Intraoperative CGs and postoperative component position

**Figure 2:**
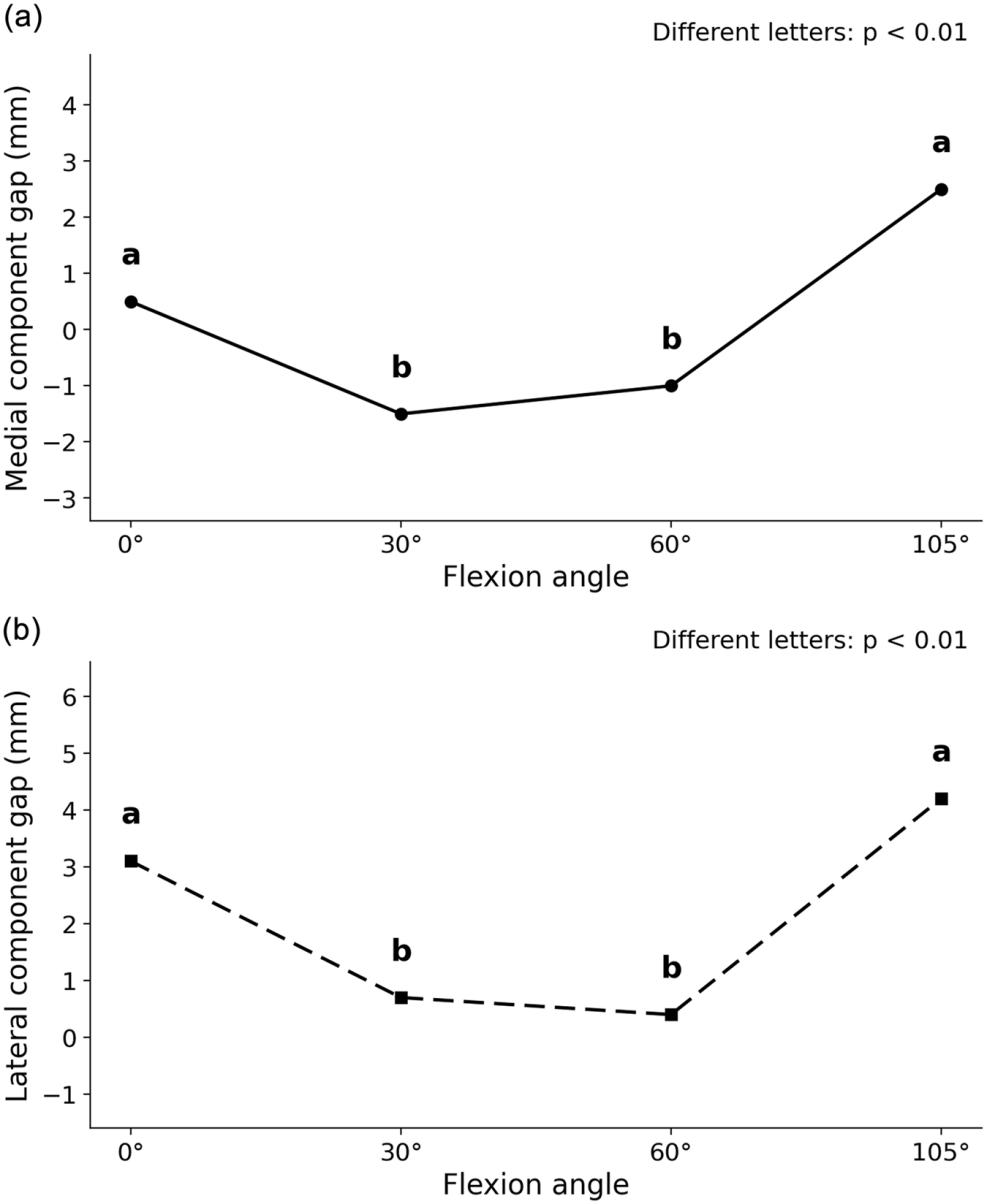
Medial and lateral component gaps at each flexion angle. (a) Medial CG at 0°, 30°, 60° and 105° of flexion. (b) Lateral CG at 0°, 30°, 60° and 105° of flexion. Values are presented as mean ± standard deviation. Negative values indicate a tighter gap, and positive values indicate a looser gap. Different letters indicate significant differences (*p* < 0.01).

The mean medial CG values were 0.5 ± 1.4 mm (range, −2.7 to 4.0 mm) at 0° flexion, −1.5 ± 1.8 mm (range, −6.7 to 3.0 mm) at 30° flexion, −1.0 ± 2.1 mm (range, −5.3 to 4.3 mm) at 60° flexion and 2.5 ± 2.1 mm (range, −3.0 to 7.0 mm) at 105° flexion. The corresponding lateral CG values were 3.1 ± 1.6 mm (range, −0.4 to 6.3 mm), 0.7 ± 1.9 mm (range, −3.0 to 6.0 mm), 0.4 ± 2.3 mm (range, −3.7 to 8.6 mm) and 4.2 ± 2.1 mm (range, −0.7 to 9.0 mm), respectively. In both compartments, the CGs at 30° and 60° were significantly smaller than those at 0° and 105° (Figure 2). Postoperative component alignment and the corresponding intraoperative planned alignment are summarised in Table 3. A planned value for tibial rotational alignment was not defined because tibial component rotation was determined intraoperatively according to surgical findings, with the range‐of‐motion (ROM) technique serving as an adjunctive reference.

**Table 3:** Component alignment and positioning accuracy.

Variable	Intraoperative planned	Postoperative position	Mean difference	Absolute error
Femoral coronal alignment (valgus +, varus –), °	−1.1 ± 1.2 (range, −3.0 to 3.0)	−1.9 ± 1.8 (range, −10.8 to 3.0)	−0.8 ± 1.4 (range, −8.8 to 2.0)	1.1 ± 1.1 (range, 0.0–8.8)
Femoral sagittal alignment (extension +, flexion –), °	−5.0 ± 1.2 (range, −7.0 to −3.0)	−3.0 ± 2.1 (range, −7.8 to 4.1)	2.0 ± 1.9 (range, −3.0 to 10.1)	2.3 ± 1.6 (range, 0.0 to 10.1)
Femoral rotational alignment (external +, internal –), °	1.8 ± 1.2 (range, −1.0 to 4.0)	2.3 ± 1.8 (range, −1.5 to 8.5)	0.5 ± 1.8 (range, −3.5 to 5.6)	1.5 ± 1.2 (range, 0.0–5.6)
Tibial coronal alignment (valgus +, varus –), °	0.0 ± 0.0 (range, 0.0–0.0)	0.2 ± 1.0 (range, −2.7 to 3.4)	0.2 ± 1.0 (range, −2.7 to 3.4)	0.7 ± 0.7 (range, 0.0–3.4)
Tibial sagittal alignment (extension +, flexion –), °	−3.5 ± 0.6 (range, −4.0 to 0.0)	−2.1 ± 1.6 (range, −6.1 to 2.5)	1.3 ± 1.5 (range, −2.5 to 5.9)	1.6 ± 1.2 (range, 0.0–5.9)
Tibial rotational alignment (external +, internal –), °	‐	−7.8 ± 6.2 (range, −21.5 to 5.1)	‐	‐

*Note*: Values are expressed as mean ± standard deviation. Coronal alignment was defined as positive for valgus and negative for varus, sagittal alignment as positive for extension and negative for flexion and rotational alignment as positive for external rotation and negative for internal rotation.

### Associations between each CG and postoperative clinical outcomes

**Figure 3:**
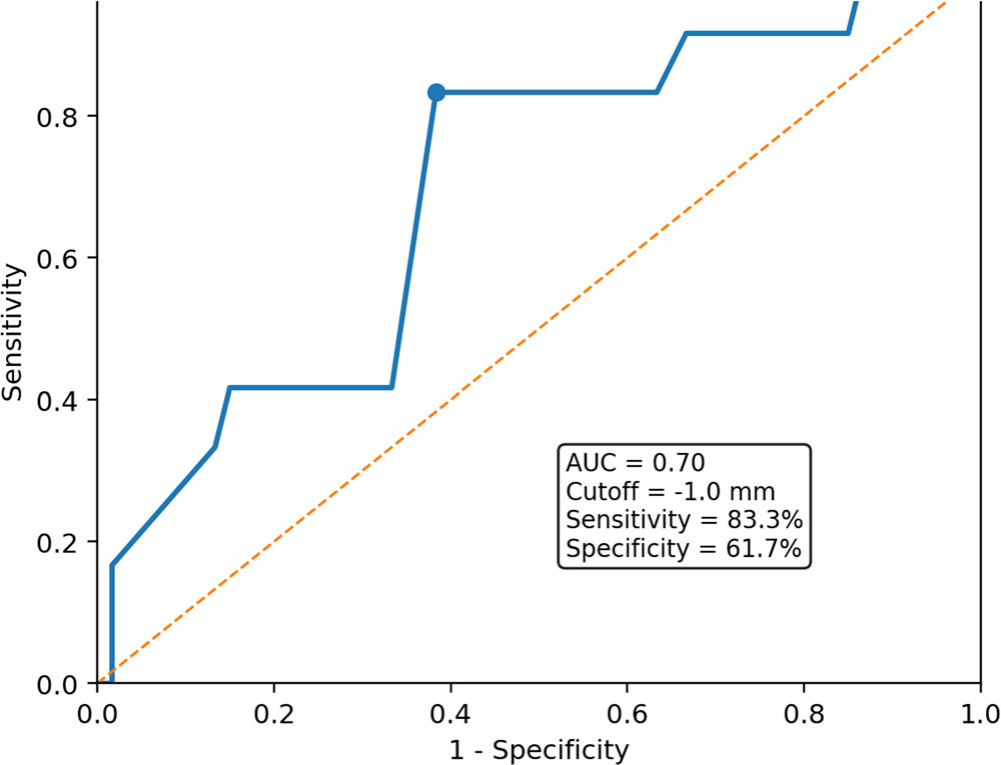
Receiver operating characteristic curve of Med CG30° for SRKI at 1 year postoperatively. In ROC analysis, the AUC of Med CG30° for SRKI at 1 year postoperatively was 0.70, and the optimal cutoff value was −1.0 mm. Sensitivity was 83.3% and specificity was 61.7%. AUC, area under the curve; CG, component gap; ROC, receiver operating characteristic; SRKI, self‐reported knee instability.

When the associations between CG at each flexion angle and in each compartment and postoperative clinical outcomes were examined, Med CG30° showed the most consistent association with postoperative outcomes (Table 4). Increased Med CG30° was significantly associated with SRKI positivity at 1 year (OR 1.73 per 1‐mm increase; 95% CI: 1.09–2.74; *p* = 0.020) and with a lower KOOS total score (*β* = −1.69 per 1‐mm increase; 95% CI: − 3.32 to −0.06; *p* = 0.043). In contrast, no similarly consistent associations were observed for the other flexion angles or for the lateral CGs. In ROC analysis, the AUC of Med CG30° for SRKI at 1 year was 0.70 and the optimal cutoff value was −1.0 mm (Figure 3). Using the ROC‐derived cutoff value of −1.0 mm for Med CG30°, 33 of 72 knees (45.8%) were classified as having increased medial component laxity.

**Table 4:** Associations of each CG with SRKI and KOOS total at 1 year postoperatively.

Predictor	SRKI at 1 year OR per 1 mm	95% CI	*p‐*Value	KOOS total *β* per 1 mm	95% CI	*p‐*Value
Medial CG at 0° flexion	1.32	0.76–2.27	0.325	0.49	−1.64 to 2.61	0.650
Medial CG at 30° flexion	1.73	1.09–2.74	0.020	−1.69	−3.32 to −0.06	0.043
Medial CG at 60° flexion	1.34	0.98–1.85	0.070	−0.77	−2.18 to 0.63	0.274
Medial CG at 105° flexion	1.27	0.92–1.76	0.146	−1.70	−3.04 to −0.35	0.014
Lateral CG at 0° flexion	1.08	0.70–1.66	0.739	1.47	−0.28 to 3.21	0.098
Lateral CG at 30° flexion	1.21	0.84–1.76	0.307	0.54	−0.98 to 2.06	0.482
Lateral CG at 60° flexion	1.22	0.92–1.62	0.158	0.00	−1.28 to 1.29	0.995
Lateral CG at 105° flexion	0.97	0.72–1.31	0.826	0.26	−1.14 to 1.65	0.714

*Note*: Odds ratios and regression coefficients are shown per 1‐mm increase in CG. Logistic regression analysis was used for SRKI and linear regression analysis for KOOS total. All models were adjusted for age and BMI. A negative β coefficient indicates a lower postoperative KOOS total score with increasing CG.

Abbreviations: CG, component gap; CI, confidence interval; KOOS, knee injury and osteoarthritis outcome score; OR, odds ratio; SRKI, self‐reported knee instability.

### Analysis of component‐positioning factors associated with Med CG30°

When component‐positioning factors associated with Med CG30° were examined, femoral positioning factors were mainly associated with Med CG30° in univariable analysis. In multivariable analysis, greater femoral valgus alignment (*β* = 0.25; 95% CI: 0.00–0.50; *p* = 0.049), femoral extension (*β* = −0.25; 95% CI: − 0.44 to −0.05; *p* = 0.014) and femoral external rotation (*β* = 0.34; 95% CI: 0.09–0.59; *p* = 0.009) were independently associated with increased Med CG30°, whereas none of the tibial positioning factors showed significant associations (Table 5).

**Table 5:** Univariable and multivariable analyses of component‐positioning factors associated with Med CG30°.

Predictor	Univariable *β* per 1°	95% CI	*p‐*Value	Multivariable adjusted *β* per 1°	95% CI	*p‐*Value
Femoral coronal alignment	0.20	−0.04 to 0.45	0.106	0.25	0.00–0.50	0.049
Femoral sagittal alignment	−0.19	−0.40 to 0.01	0.056	−0.25	−0.44 to −0.05	0.014
Femoral rotational alignment	0.28	0.04–0.52	0.023	0.34	0.09– 0.59	0.009
Tibial coronal alignment	−0.01	−0.44 to 0.43	0.981	0.01	−0.44 to 0.46	0.952
Tibial sagittal alignment	−0.07	−0.34 to 0.20	0.611	0.10	−0.18 to 0.37	0.488
Tibial rotational alignment	−0.02	−0.09 to 0.05	0.574	−0.04	−0.10 to 0.03	0.282

*Note*: Med CG30° indicates the medial component gap at 30° of flexion. Regression coefficients indicate the change in Med CG30° per 1° increase in each positioning angle. The multivariable model simultaneously included femoral and tibial coronal, sagittal and rotational alignment.

Abbreviations: CI, confidence interval; Med CG30°, medial component gap at 30° flexion.

## DISCUSSION

The hypothesis of this study was supported. The most important finding of this study was that increased Med CG30° was associated with postoperative subjective instability and worse patient‐reported outcomes after image‐free rTKA. Specifically, an increase in Med CG30° was associated with SRKI positivity at 1 year and with a lower KOOS total score. A previous study reported that, in image‐free rTKA using the Journey II implant, the CG in the mid‐flexion range was smaller than that in extension and deep flexion and might contribute to stabilisation in the mid‐flexion range [20]. The present study extends those findings by demonstrating that the looseness of Med CG30° is actually related to postoperative patient‐reported clinical outcomes.

Several previous studies have focused on laxity in the mid‐flexion range and its association with clinical outcomes. Hasegawa et al. reported that intraoperative mid‐flexion medial laxity measured using navigation in posterior‐stabilised TKA was associated with lower patient expectations [8]. Inui et al. reported that increased medial laxity at 30° of flexion in bi‐cruciate stabilised TKA was associated with lower patient satisfaction [13]. Mochizuki et al. also reported that anteroposterior laxity in the mid‐range of flexion was associated with subjective postoperative instability [25], suggesting that instability in the mid‐flexion range may directly translate into patient discomfort and a subjective feeling of instability. These reports are consistent with the present finding that Med CG30° showed the most consistent association with postoperative outcomes. The present results suggest that, in soft‐tissue balance assessment during TKA, attention should be paid not only to balance in extension and flexion but also to Med CG30°. Although the concept of MFI has been reported previously, the present study provides novel evidence that quantitatively measured Med CG30° during image‐free rTKA is associated with postoperative subjective instability and patient‐reported outcomes.

Furthermore, ROC analysis showed that the AUC of Med CG30° for SRKI at 1 year was 0.70 and that the optimal cutoff value was −1.0 mm. Knees with Med CG30° looser than −1.0 mm may therefore be at higher risk of postoperative subjective instability. Although the AUC was not particularly high, postoperative instability is unlikely to be determined by a single intraoperative factor alone, and Med CG30° may still serve as a clinically useful indicator with moderate discriminative ability for predicting postoperative instability. The cutoff value of −1.0 mm should be interpreted as the optimal threshold identified by ROC analysis in patients undergoing TKA with the Journey II Bi‐Cruciate Stabilised implant rather than as a definition of medial tightness.

In recent years, multiple alignment concepts for TKA have been proposed, including mechanical alignment, kinematic alignment and anatomic alignment. However, the value of rTKA may lie not only in improving bony resection accuracy [1] but also in enabling optimisation of component position while taking into account quantitative intraoperative gap information throughout the range of motion, including the mid‐flexion range [2, 27].

Recent studies have further emphasised the importance of quantitative intraoperative gap assessment and knee laxity evaluation during rTKA. Kaneko et al. reported that intraoperative gap characteristics may influence postoperative patient‐reported outcomes following robotic‐assisted TKA [14]. In addition, Kenanidis et al. proposed the robotic evaluation of articular laxity (REAL) classification, highlighting the growing importance of quantitative intraoperative knee laxity assessment and characterisation of knee balance phenotypes during robotic‐assisted TKA [15]. These findings support the concept that quantitative assessment of knee balance throughout the range of motion may provide clinically meaningful information beyond conventional extension and flexion gap evaluation. The present findings suggest that, in addition to the gap balance traditionally assessed mainly in extension and flexion, Med CG30° is also an important parameter. Med CG30° was associated with femoral component‐positioning angles, but in the surgical technique used in this study, fine adjustment for soft‐tissue balance was primarily performed on the femoral side, whereas tibial component position was essentially fixed. Therefore, this association likely reflects the procedure‐specific adjustment strategy. An important clinical implication of the present study is that excessive medial laxity at 30° of flexion should be avoided when optimising gap balance during rTKA. Because an increased Med CG30° was associated with postoperative subjective instability and worse patient‐reported outcomes, surgeons should carefully assess Med CG30° during intraoperative gap balancing. Based on these findings, when component position is adjusted to optimise gap balance, care should be taken to avoid excessive looseness of Med CG30°, particularly when increasing femoral valgus, extension or external rotation. However, because this relationship was observed using the balancing strategy used in this study, in which tibial component position was maintained and gap balance was optimised primarily through femoral component adjustment, further studies are required to determine whether similar findings apply to other alignment philosophies and balancing strategies.

This study has several limitations. First, it was a single‐centre study with a relatively small sample size, and caution is required in generalising the results. Although patients were consecutively enrolled and no eligible patients declined participation, 29 of 101 enrolled knees were excluded from the final analysis, which may have introduced selection bias. Second, the analysis was limited to image‐free rTKA using the Journey II implant, and the findings may not be directly applicable to other implant designs or alignment strategies. Third, postoperative outcomes were evaluated only at 1 year, and their relationship with longer‐term clinical outcomes remains unclear. In addition, because this was an observational study, it cannot directly prove a causal relationship between looseness of Med CG30° and postoperative outcomes. Nevertheless, the study is meaningful in that it compared CGs across multiple flexion angles and compartments and demonstrated the clinical importance of Med CG30° and the relevance of its management.

In summary, in image‐free rTKA using the Journey II system, an increase in Med CG30° was associated with poorer postoperative outcomes, suggesting that careful management of this gap is important.

## CONCLUSION

In image‐free rTKA using the Journey II system, an increased Med CG30° was associated with postoperative subjective instability and worse patient‐reported clinical outcomes. These findings suggest that, in soft‐tissue balance assessment during TKA, attention should be paid not only to the extension and flexion gaps but also to Med CG30°, and that its management is important.

## AUTHOR CONTRIBUTIONS

Tomoharu Mochizuki and Keisuke Maeda conceived and designed the study. Keisuke Maeda, Tomoharu Mochizuki, Keiichiro Someya, Yutaka Fujita, Yuya Majima, Shigeru Takagi, Go Omori and Hiroyuki Kawashima acquired the data. Keisuke Maeda and Tomoharu Mochizuki analysed and interpreted the data. Keisuke Maeda drafted the manuscript. Tomoharu Mochizuki critically revised the manuscript. All authors approved the final manuscript.

## CONFLICT OF INTEREST STATEMENT

The authors declare no conflicts of interest.

## FUNDING INFORMATION

The authors have no funding to report.

## ETHICS STATEMENT

All procedures performed in this study involving human participants were in accordance with the ethical standards of the institutional research committee and with the 1964 Declaration of Helsinki and its later amendments or comparable ethical standards. This prospective observational study was approved by the ethics committee of Niigata University (Approval No. 2020‐0448). Written informed consent was obtained from all participants.

## Data Availability

The datasets generated and/or analysed during the current study are not publicly available due to institutional restrictions regarding patient data but are available from the corresponding author on reasonable request.

## References

[1] Alton TB , Severson EP , Ford MC , Lesko J , Leslie IJ . VELYS robotic‐assisted total knee arthroplasty: enhanced accuracy and comparable early outcomes versus manual instrumentation during adoption. J Exp Orthop. 2025;12:e70163. 39931150 10.1002/jeo2.70163 PMC11808256

[2] Andriollo L , Diquattro E , Koutserimpas C , Mazzella GG , Bonat G , Servien E , et al. Robotic total knee arthroplasty with functional positioning safely addresses major coronal deformities: comparable complications and survivorship. J Exp Orthop. 2026;13:e70613. 41531476 10.1002/jeo2.70613 PMC12794669

[3] Baier C , Wolfsteiner J , Otto F , Zeman F , Renkawitz T , Springorum HR , et al. Clinical, radiological and survivorship results after ten years comparing navigated and conventional total knee arthroplasty: a matched‐pair analysis. Int Orthop. 2017;41:2037–2044. 28550426 10.1007/s00264-017-3509-z

[4] Bollars P , Boeckxstaens A , Mievis J , Kalaai S , Schotanus MGM , Janssen D . Preliminary experience with an image‐free handheld robot for total knee arthroplasty: 77 cases compared with a matched control group. Eur J Orthop Surg Traumatol. 2020;30:723–729. 31950265 10.1007/s00590-020-02624-3

[5] Clary CW , Fitzpatrick CK , Maletsky LP , Rullkoetter PJ . The influence of total knee arthroplasty geometry on mid‐flexion stability: an experimental and finite element study. J Biomech. 2013;46:1351–1357. 23499227 10.1016/j.jbiomech.2013.01.025

[6] Collins K , Agius PA , Fraval A , Petterwood J . Initial experience with the NAVIO robotic‐assisted total knee replacement‐coronal alignment accuracy and the learning curve. J Knee Surg. 2022;35:1295–1300. 33511584 10.1055/s-0040-1722693

[7] van der Esch M , Knoop J , van der Leeden M , Voorneman R , Gerritsen M , Reiding D , et al. Self‐reported knee instability and activity limitations in patients with knee osteoarthritis: results of the Amsterdam osteoarthritis cohort. Clin Rheumatol. 2012;31:1505–1510. 22729472 10.1007/s10067-012-2025-1

[8] Hasegawa M , Tone S , Naito Y , Sudo A . Intraoperative midflexion medial laxity using navigation affects patient expectations following posterior stabilized total knee arthroplasty. J Orthop Surg. 2022;30:10225536221119512. 10.1177/10225536221119512 37583311

[9] Heck DA , Melfi CA , Mamlin LA , Katz BP , Arthur DS , Dittus RS , et al. Revision rates after knee replacement in the United States. Med Care. 1998;36:661–669. 9596057 10.1097/00005650-199805000-00006

[10] Hetaimish BM , Khan MM , Simunovic N , Al‐Harbi HH , Bhandari M , Zalzal PK . Meta‐analysis of navigation vs conventional total knee arthroplasty. J Arthroplasty. 2012;27:1177–1182. 22333865 10.1016/j.arth.2011.12.028

[11] Hino K , Ishimaru M , Iseki Y , Watanabe S , Onishi Y , Miura H . Mid‐flexion laxity is greater after posterior‐stabilised total knee replacement than with cruciate‐retaining procedures: a computer navigation study. Bone Jt J. 2013;95–B:493–497. 10.1302/0301-620X.95B4.30664 23539701

[12] Inui H , Taketomi S , Yamagami R , Kawaguchi K , Nakazato K , Tanaka S . The relationship between anteroposterior stability and medial‐lateral stability of the bi‐cruciate stabilized total knee arthroplasty. Knee. 2018;25:1247–1253. 30414789 10.1016/j.knee.2018.08.002

[13] Inui H , Taketomi S , Yamagami R , Kono K , Kawaguchi K , Uehara K , et al. Influence of surgical factors on patient satisfaction after bi‐cruciate stabilized total knee arthroplasty: retrospective examination using multiple regression analysis. BMC Musculoskelet Disord. 2021;22:215. 33622292 10.1186/s12891-021-04098-8 PMC7903778

[14] Kaneko T , Shiga K , Mishima Y . Intraoperative gap assessment in robotic‐assisted bicruciate retaining TKA for knee osteoarthritis. Sci Rep. 2025;15:15675. 40325159 10.1038/s41598-025-99872-2 PMC12052840

[15] Kenanidis E , Milonakis N , Maslaris A , Tsiridis E . Robotic evaluation of articular laxity (REAL) classification: a new intraoperative knee soft‐tissue laxity classification using ROSA robotic software. Eur J Orthop Surg Traumatol. 2025;35:139. 40156716 10.1007/s00590-025-04265-w PMC11954700

[16] Kim YH , Park JW , Kim JS . The long‐term results of simultaneous high‐flexion mobile‐bearing and fixed‐bearing total knee arthroplasties performed in the same patients. J Arthroplasty. 2019;34:501–507. 30503307 10.1016/j.arth.2018.11.007

[17] Le DH , Goodman SB , Maloney WJ , Huddleston JI . Current modes of failure in TKA: infection, instability, and stiffness predominate. Clin Orthop Relat Res. 2014;472:2197–2200. 24615421 10.1007/s11999-014-3540-y PMC4048402

[18] Lombardi Jr. AV , Berend KR , Adams JB . Why knee replacements fail in 2013: patient, surgeon, or implant? Bone Jt J. 2014;96–B:101–104. 10.1302/0301-620X.96B11.34350 25381419

[19] Luyckx T , Vandenneucker H , Ing LS , Vereecke E , Ing AV , Victor J . Raising the joint line in TKA is associated with mid flexion laxity: a study in cadaver knees. Clin Orthop Relat Res. 2018;476:601–611. 29443845 10.1007/s11999.0000000000000067 PMC6260050

[20] Maeda K , Mochizuki T , Takagi S , Omori G , Yamamoto N , Kobayashi K , et al. Image‐free robotic‐assisted total knee arthroplasty is associated with joint line distalization and improves mid‐flexion instability: a prospective cohort study. J Exp Orthop. 2025;12:e70239. 40226533 10.1002/jeo2.70239 PMC11993982

[21] Martin JW , Whiteside LA . The influence of joint line position on knee stability after condylar knee arthroplasty. Clin Orthop Relat Res. 1990;259:146–156. 2208849

[22] Matsumoto K , Ogawa H , Yoshioka H , Akiyama H . Postoperative anteroposterior laxity influences subjective outcome after total knee arthroplasty. J Arthroplasty. 2017;32:1845–1849. 28143687 10.1016/j.arth.2016.12.043

[23] Matsumoto T , Nakano N , Hayashi S , Takayama K , Maeda T , Ishida K , et al. Prosthetic orientation, limb alignment, and soft tissue balance with bicruciate stabilized total knee arthroplasty: a comparison between the handheld robot and conventional techniques. Int Orthop. 2023;47:1473–1480. 36928553 10.1007/s00264-023-05737-6 PMC10199853

[24] McCalden RW , Hart GP , MacDonald SJ , Naudie DD , Howard JH , Bourne RB . Clinical results and survivorship of the GENESIS II total knee arthroplasty at a minimum of 15 years. J Arthroplasty. 2017;32:2161–2166. 28285899 10.1016/j.arth.2017.02.006

[25] Mochizuki T , Tanifuji O , Sato T , Hijikata H , Koga H , Watanabe S , et al. Association between anteroposterior laxity in mid‐range flexion and subjective healing of instability after total knee arthroplasty. Knee Surg Sports Traumatol Arthrosc. 2017;25:3543–3548. 27830283 10.1007/s00167-016-4375-8

[26] Roos EM , Roos HP , Lohmander LS , Ekdahl C , Beynnon BD . Knee injury and osteoarthritis outcome score (KOOS): development of a self‐administered outcome measure. J Orthop Sports Phys Ther. 1998;28:88–96. 9699158 10.2519/jospt.1998.28.2.88

[27] Sacco R , Tecame A , Lalevée M , Perrier A , Massa E , Kouyoumdjian P , et al. Robotic vs. conventional total knee arthroplasty over two decades: evolving trends toward personalised alignment without significant clinical superiority in predominantly mild varus deformity—a systematic review of RCTs. J Exp Orthop. 2025;12:e70452. 41341124 10.1002/jeo2.70452 PMC12670295

[28] Sato T , Koga Y , Omori G . Three‐dimensional lower extremity alignment assessment system. J Arthroplasty. 2004;19:620–628. 15284984 10.1016/j.arth.2003.12.063

[29] Vince KG . Why knees fail. J Arthroplasty. 2003;18:39–44. 12730927 10.1054/arth.2003.50102

